# Chemical‐induced craniofacial anomalies caused by disruption of neural crest cell development in a zebrafish model

**DOI:** 10.1002/dvdy.179

**Published:** 2020-05-05

**Authors:** Shujie Liu, Rika Narumi, Naohiro Ikeda, Osamu Morita, Junichi Tasaki

**Affiliations:** ^1^ R&D, Safety Science Research, Kao Corporation Tochigi Japan

**Keywords:** disease model, environmental factors, neural crest cells, neurocristopathy, teratogen, zebrafish

## Abstract

**Background:**

Craniofacial anomalies are among the most frequent birth defects worldwide, and are thought to be caused by gene‐environment interactions. Genetically manipulated zebrafish simulate human diseases and provide great advantages for investigating the etiology and pathology of craniofacial anomalies. Although substantial advances have been made in understanding genetic factors causing craniofacial disorders, limited information about the etiology by which environmental factors, such as teratogens, induce craniofacial anomalies is available in zebrafish.

**Results:**

Zebrafish embryos displayed craniofacial malformations after teratogen treatments. Further observations revealed characteristic disruption of chondrocyte number, shape and stacking. These findings suggested aberrant development of cranial neural crest (CNC) cells, which was confirmed by gene expression analysis of the CNC. Notably, these observations suggested conserved etiological pathways between zebrafish and mammals including human. Furthermore, several of these chemicals caused malformations of the eyes, otic vesicle, and/or heart, representing a phenocopy of neurocristopathy, and these chemicals altered the expression levels of the responsible genes.

**Conclusions:**

Our results demonstrate that chemical‐induced craniofacial malformation is caused by aberrant development of neural crest. This study indicates that zebrafish provide a platform for investigating contributions of environmental factors as causative agents of craniofacial anomalies and neurocristopathy.

## 1 INTRODUCTION

Craniofacial anomalies represent a diverse group of deformities related to the differentiation and growth of the head and facial bones, and comprise over one‐third of all congenital birth defects.^1^ According to the OMIM database (https://www.ncbi.nlm.nih.gov/omim), over 700 disorders are associated with craniofacial features. The most frequent craniofacial anomalies comprise orofacial clefts, cleft lip, and/or cleft palate.^2^ In addition, craniosynostosis, hemifacial microsomia, and holoprosencephaly are other common symptoms. These malformations are closely linked to neural crest (NC) cell development, with defects in the formation, migration, and differentiation of NC cells, which are a cell population formed in vertebrates during embryogenesis.^3^, ^4^ These cells are destined to differentiate into various cell types, such as neurons, glia, chondrocytes, melanocytes, and smooth muscle myocytes.^5^, ^6^, ^7^ Thus, the disruption of NC cell development leads to diverse clinical pathologies, collectively termed neurocristopathy.^8^, ^9^, ^10^ These malformations are thought to be caused by genetic mutations in specific genes. Several clinical syndromes involving neurocristopathy, including Treacher Collins syndrome (OMIM: #154500), CHARGE syndrome (OMIM: #214800), Pierre Robin sequence (OMIN: #261800), Branchio‐oculo‐facial syndrome (OMIN: #113620), and Waardenburg syndrome (OMIM: #193500), are well characterized phenotypically and genetically.^11^, ^12^, ^13^, ^14^ In addition to genetic mutations, environmental factors such as alcohol, folic acid deficiency, maternal diabetes, infection, and pharmaceutical agents have been reported to be involved in craniofacial anomalies.^15^, ^16^, ^17^ Therefore, these abnormalities are believed to be induced by genetic and environmental factors.^18^, ^19^, ^20^, ^21^ As an example of gene‐environment interaction, there is an increased risk of the development of orofacial clefts in carriers of a specific genotype if their mothers smoke cigarettes during pregnancy.^22^ However, the causal relationship between environmental and genetic factors in craniofacial malformations induced by pharmaceuticals and chemical agents remains generally unknown, despite the existence of such a relationship being widely accepted. Furthermore, while identifying environmental factors that cause chemical‐induced malformations and their degree of risk has advanced as a major goal for preventing congenital birth defects, the underlying cellular and molecular mechanisms remain poorly understood due to the lack of suitable model systems.

As one model system, zebrafish (*Danio rerio*) have been used to examine the etiology of human craniofacial anomalies.^23^, ^24^, ^25^, ^26^, ^27^ Zebrafish have a simple craniofacial structure, and facial morphogenesis is easily observed due to the embryo's transparency after external fertilization. Several responsible genes whose malfunction induces craniofacial malformations have been identified and are conserved between human and zebrafish, including *Tcof1* and *Polr1* (Treacher Collins syndrome), *Chd7* (CHARGE syndrome), *Sox9* (Pierre Robin sequence), *Tfap2a* (Branchio‐Oculo‐Facial syndrome), and *Sox10* (Waardenburg syndrome).^28^, ^29^, ^30^, ^31^, ^32^, ^33^ In addition, genetic modifications of the disease‐related genes in zebrafish simulate the clinical phenotypes found in humans.^28^, ^29^, ^30^, ^31^, ^32^, ^33^ This evidence supports the hypothesis that zebrafish provide a novel platform for analyzing gene‐environment interactions that affect craniofacial malformations. Some knowledge has been accumulated regarding the association between genotypes and phenotypes in human craniofacial anomalies by utilizing the zebrafish model. Fetal alcohol spectrum disorder (FASD) is a well‐known example of gene environment interaction and zebrafish FASD model is a powerful model for analyzing prenatal exposure of alcohol.^34^, ^35^, ^36^ There are only a few reports except reports using the FASD model that have focused on the cellular and molecular mechanisms underlying chemical‐induced craniofacial malformations.

In short, there are two questions to be addressed: (a) Do teratogens which induce craniofacial anomalies in mammals cause the same anomalies in zebrafish? (b) Do teratogens target CNC development to induce craniofacial anomalies in zebrafish?

In the present study, zebrafish embryos were treated with 12 chemicals (retinoic acid, methotrexate, salicylic acid, valproic acid, caffeine, warfarin, hydroxyurea, dexamethasone, phenytoin, imatinib, boric acid, and thalidomide) which are recognized to be teratogens in mammals. Prominent morphological defects were found in the craniofacial region and heart, which strongly suggested aberrant development of NC cells. The craniofacial morphology of the teratogen‐treated embryos was examined by whole‐mount cartilage staining. All of these zebrafish displayed malformations of the neurocranium and viscerocranium, which are composed of NC‐derived chondrocytes. In particular, a smaller lower jaw (micrognathia) and anterior neurocranial defects appeared to be due to a decreased number of chondrocytes and defects related to the morphologies of chondrocytes. Next, the expression levels of genes regulating cranial neural crest (CNC) development were examined. These gene expression levels were found to be decreased, further suggesting the disruption of CNC development. Our results indicated that CNC development is a target biological pathway whose dysregulation underlies chemical‐induced craniofacial malformation. Interestingly, the expression levels of genes responsible for specific neurocristopathies also decreased, suggesting that the zebrafish phenotype resembles neurocristopathy phenotypically.

Taken together, our findings suggest that chemical‐induced craniofacial malformation is caused by a defect of CNC development, which is the same etiology as that of various clinical symptoms resulting from genetic mutations. Our results also indicate that zebrafish would be a novel platform to identify and examine the contribution of environmental factors to craniofacial malformation.

## 2 RESULTS

### 2.1 Teratogen exposure induced abnormal craniofacial development in zebrafish

**FIGURE 1:**
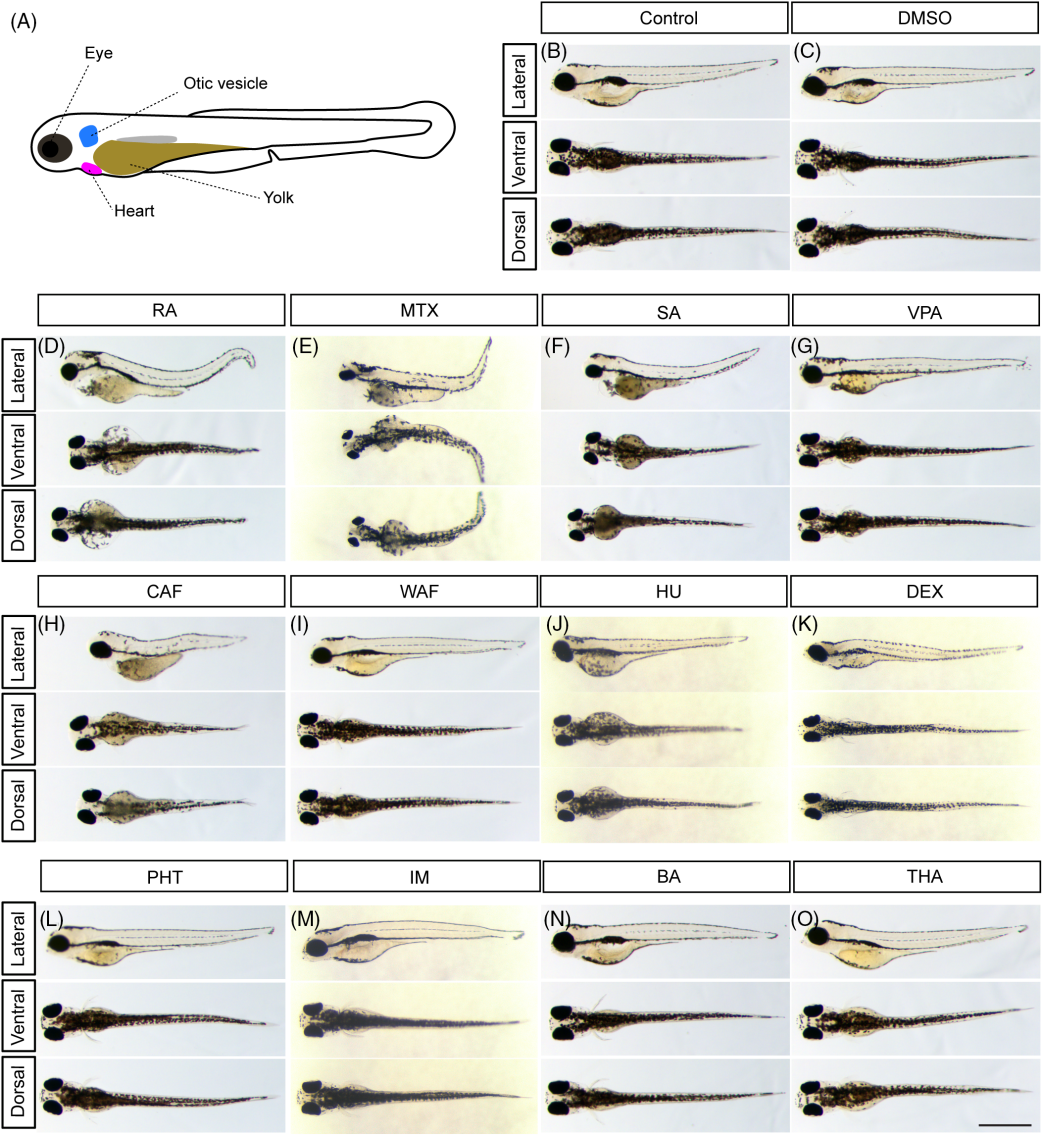
Gross morphological anomalies of teratogen‐treated zebrafish embryos at 96 hpf. A, Schematic diagram of zebrafish embryo at 96 hpf. B, E3 control. C, DMSO control. D, RA, retinoic acid. E, MTX, methotrexate. F, SA, salicylic acid. G, VPA, valproic acid. H, CAF, caffeine. I, WAF, warfarin. J, HU, hydroxyurea. K, DEX, dexamethasone. L, PHT, phenytoin. M, IM, imatinib. N, BA, boric acid. O, THA, thalidomide. Scale bar: 1 mm

**FIGURE 2:**
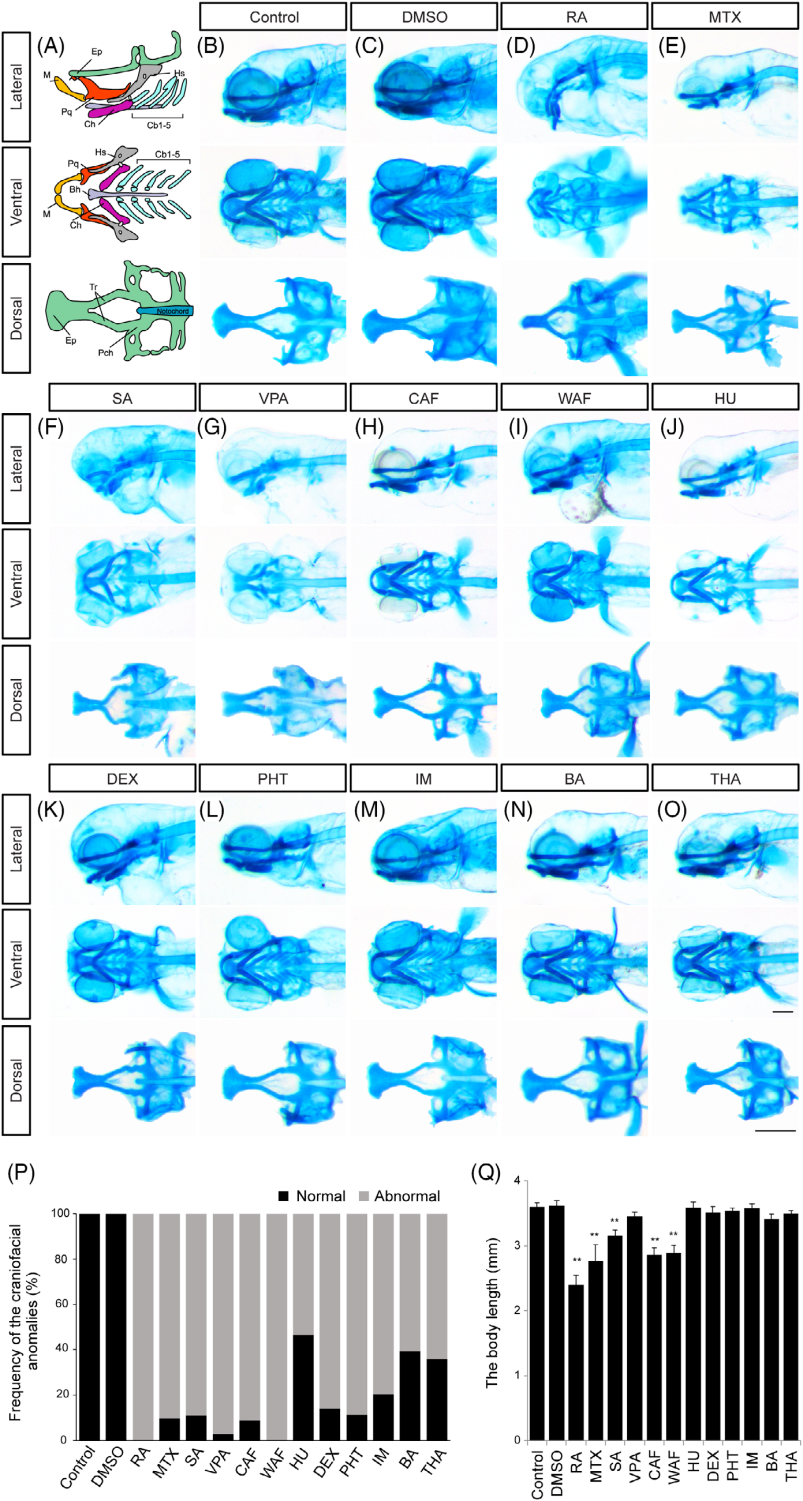
Alcian blue‐stained zebrafish embryos at 96 hpf displayed abnormal cranial development. A, Craniofacial atlas of the lateral view, the viscerocranium (ventral view) and the neurocranium (dorsal view): Bh, Basihyal; Cb, Ceratobranchial; Ch, Ceratohyal; Hm, Hyomandibula; Hs, Hyosymplectic; Ih, Interhyal; M, Meckel's; OP, Opercle (bone); Pq, Palatoquadrate; Ep, Ethmoid plate; Tr, Trabeculae; Pch, Parachordal of craniofacial structures. B‐O, Zebrafish treated with the following teratogens showed cranial malformations: B, Control, E3; C, DMSO, vehicle control; D, RA, retinoic acid; E, MTX, methotrexate; F, SA, salicylic acid; G, VPA, valproic acid; H, CAF, caffeine; I, WAF, warfarin; J, HU, hydroxyurea; K, DEX, dexamethasone; L, PHT, phenytoin; M, IM, imatinib; N, BA, boric acid; O, THA, thalidomide. P, The summary of craniofacial anomalies (Control: n = 45, DMSO: n = 40, RA: n = 35, MTX: n = 31, SA: n = 27, VPA: n = 36, CAF: n = 34, WAF: n = 32, HU: n = 28, DEX: n = 36, PHT: n = 35, IM: n = 27, BA: n = 33, THA: n = 39). Q, The body length analysis. Scale bars: 200 μm

To investigate the effects of teratogens on zebrafish craniofacial development, embryos were exposed to the 12 teratogens listed in Table 1. These teratogens are known to induce craniofacial defects such as cleft palate, micrognathia, ear anomalies and eye defects in mammals. Morphological changes of the embryos at 96 hours post fertilization (hpf) were examined in the head, eyes and otic vesicles (Figure 1). Although embryos treated with five chemicals (RA, MTX, SA, CAF, and HU) displayed gross morphological defects, embryos treated with the other chemicals showed only minor phenotypes due to the embryos' transparency (Figure 1B–O). Therefore, for better visualization of the cranial structure and its phenotype, cartilage staining with Alcian blue was performed (Figure 2). The cartilaginous head skeleton was clearly visualized at 96 hpf and consisted of two prominent subdivisions, which were the neurocranium composed of the ethmoid plate, trabeculae and parachordal, and the viscerocranium composed of the pharyngeal skeleton, such as Meckel's cartilage, palatoquadrate, ceratobranchial, and ceratohyal (Figure 2A). No phenotypic differences were detected between E3 control and DMSO as vehicle control (Figure 2B,C). Specific defects in the viscerocranium and the neurocranium were clearly detected in embryos treated with each of the teratogens tested (Figure 2D–O). RA‐, MTX‐, SA‐, and VPA‐treated embryos displayed severe defects in the neurocranium and the viscerocranium (Figure 2D–G). In these embryos, deformities of the craniofacial skeleton were marked by the absence or shortening of the skeletal elements in the viscerocranium and malformation of the ethmoid plate, which consisted of a fused (RA treatment) or separated (MTX, SA, and VPA treatment) ethmoid plate (Figure 2D–G). CAF‐, WAF‐, HU‐, and DEX‐treated embryos showed moderate phenotypes, such as shortening of Meckel's cartilage and a separate or short ethmoid plate (Figure 2H–K). PHT‐, IM‐, BA‐, and THA‐treated embryos showed slight changes in both the neurocranium and the viscerocranium (Figure 2L–O). In these embryos, the anteroposterior length of the ethmoid plate and trabeculae and the width of the parachordal were shorter than those in the control group. Clear differences in palatoquadrate, hyosymplectic, ceratohyal, and ceratobranchial cartilages were not detected; however, shortened Meckel's cartilage was observed in these embryos (Figure 2L–O). These morphological defects were quantified and summarized in Figure 2P. The dimensions of the morphological changes found in the head structure were greater than those of the changes in overall size of the embryos (Figure 2Q).

**TABLE 1:** Compounds used in the present study

Category	Test compound	Abbreviation	CAS no.	Supplier	Solvent	Concentration range (μM)	Craniofacial defects in mammals
Antineoplastic agents, immunomodulating agents	Hydroxyurea	HU	127‐07‐1	Sigma‐Aldrich	Distilled water	1–1,000	Craniofacial dysgenesis, micrognathia, cleft palate, and eye defects^37^, ^38^, ^39^
	Imatinib	IM	152 459‐95‐5	Tokyo Chemical Industry	DMSO	1–250^a^	Micrognathia, cleft palate, misshapen snouts, eye defects^40^, ^41^
	Methotrexate	MTX	59‐05‐2	Wako	DMSO	0.1–200^b^	Micrognathia, cleft lip and palate, prominent eyes, low‐set ears, and microphthalmia^42^, ^43^, ^44^
	Thalidomide	THA	50‐35‐1	Tocris Bioscience	DMSO	1–400^a^	Micrognathia, retrognathia and cleft palate, mild low set ears, and eye deformities^45^, ^46^
Cardiovascular agents antithrombotic agents, anticoagulants	Warfarin	WAF	129‐06‐6	Wako	DMSO	0.1–60^b^	Microcephaly, short palpebral fissures, maxillary hypoplasia, micrognathia, and cleft palate^47^, ^48^, ^49^, ^50^
Antiepileptic agents, anticonvulsants	Phenytoin	PHT	57‐41‐0	Wako	DMSO	1–200^a^	Micrognathia, agnathia, and cleft palate^51^, ^52^, ^53^
	Valproic acid	VPA	99‐66‐1	Wako	Distilled water	0.1–30^b^	Micrognathia, cleft palate, flattened nasal bridge, eye and ear abnormalitie^54^, ^55^, ^56^
Nonsteroidal anti‐inflammatory drugs	Salicylic acid	SA	54‐21‐7	Wako	Distilled water	1–400^b^	Micrognathia, cleft palate, and microcephaly^57^, ^58^, ^59^
Corticosteroids, immunosuppressive agents	Dexamethasone	DEX	59‐05‐2	Wako	DMSO	1–200^a^	Micrognathia and cleft palate^60^, ^61^
Ophthalmic agents, insecticide	Boric acid	BA	10043‐35‐3	Wako	Distilled water	1–1,000	Micrognathia and low‐set ears^62^, ^63^
Psychostimulants, agents used for ADHD	Caffeine	CAF	58‐08‐2	Wako	Distilled water	1–500^b^	Micrognathia, agnathia, cleft lip and palate^64^, ^65^, ^66^
Vitamin A, topical use in acne	Retinoic acid	RA	302‐79‐4	Tokyo Chemical Industry	DMSO	1 × 10^−3^ to 5 × 10^−2^ ^a^	Micrognathia, agnathia, cleft lip and palate, ear and eye defects^67^, ^68^, ^69^, ^70^

a Highest soluble concentration.

b Highest viable concentration.

**FIGURE 3:**
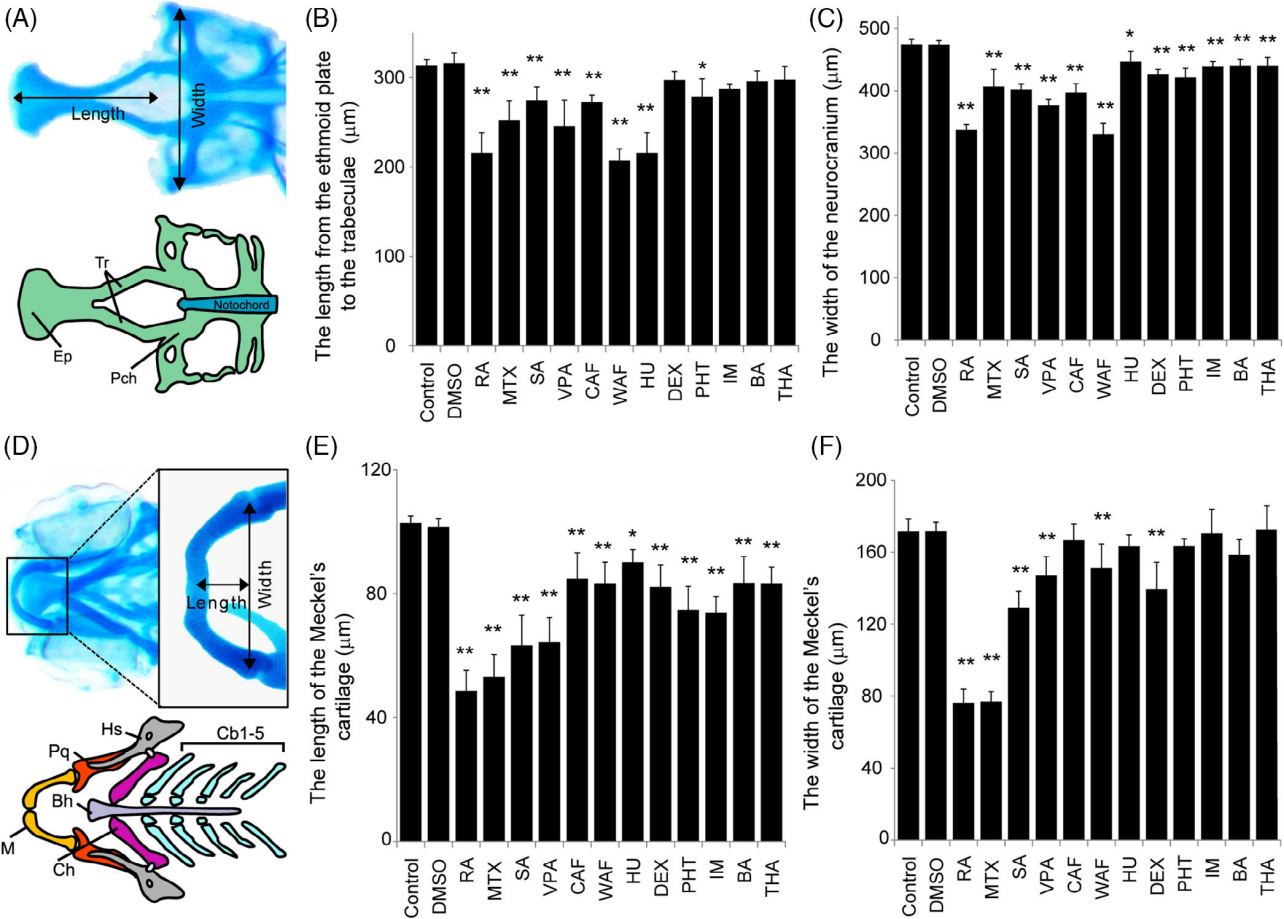
Quantitative measurement of craniofacial malformations. A, The definitions of the length and width in neurocranium measurements. B and C, The length and width of the neurocranium were quantified. D, The definition of the length and width of the Meckel's cartilage in viscerocranium measurement. E and F, The length and width of the Meckel's cartilage were quantified. The one‐way ANOVA followed by Dunnett's multiple comparison tests was used to compare the values between the control and the chemical‐treated group (*P < .05, **P < .01, n = 5)

Based on these observations, we speculated that the ethmoid plate and trabeculae in the neurocranium and Meckel's cartilage in the viscerocranium had sensitive responses to the teratogens. To test this, we then performed quantitative analysis of both the neurocranium cartilages and Meckel's cartilage (Figure 3). For the neurocranium analyses, the anteroposterior length of the ethmoid plate and trabeculae were measured and the width was measured from one side of the parachordal to the other (Figure 3A). The average neurocranium length in the teratogen‐treated embryos was significantly decreased compared to that in control embryos except DEX, IM, BA and THA (Figure 3B). Also, the average width of the neurocranium in the teratogen‐treated embryos was significantly decreased (Figure 3C). These analyses suggested that all of these teratogens affected neurocranium morphogenesis, resulting in smaller neurocranium phenotypes. Likewise, the length and width of the Meckel's cartilage in the viscerocranium were quantified (Figure 3D–F). The length was measured from the anterior edge of the Meckel's cartilage midline to the tip of the retroarticular, and the width was defined as the distance between the opposing retroarticulars (Figure 3D). The average length of the Meckel's cartilage was significantly decreased by treatment with each of the teratogens except CAF, HU, PHT IM, BA, and THA (Figure 3E). The average width of the Meckel's cartilage was decreased significantly by treatment with each of the teratogens (Figure 3F).

Thus, the sizes of the neurocranium and viscerocranium were significantly decreased by treatment with the teratogens. All of the teratogens induced small jaw (micrognathia) and small head (microcephaly), and large jaw or head or hyperplasia of the craniofacial skeleton was not observed. Together, our data suggested that the teratogens affected the craniofacial development, leading to craniofacial malformation. The ethmoid plate, trabeculae and Meckel's cartilage consist of chondrocytes, which originate from NC cells,^25^, ^71^ and therefore our findings suggest that the development of NC cells was disrupted by these teratogens.

### 2.2 Teratogen‐induced craniofacial malformations were caused by decreased chondrocyte number, and by disturbed chondrocyte shape and stacking

**FIGURE 4:**
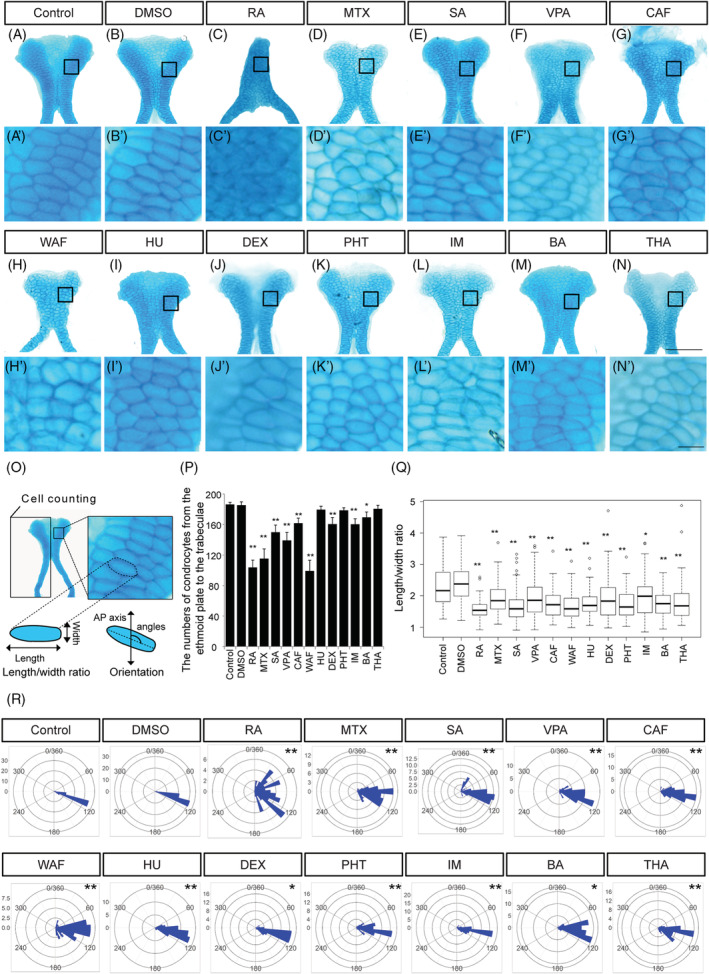
The number of chondrocytes and their shape in ethmoid plate were affected by teratogen treatment. A–N, The ethmoid plate was dissected from Alcian blue‐stained samples and was flat‐mounted. Anterior is to the top. A′–N′, Magnified view of chondrocytes in the ethmoid plate in the region indicated by the boxed area in A–N. O, The region in which cell counts were determined in the ethmoid plate and the definitions of the length and width of the chondrocytes used for the cell shape analysis. The orientation of longest cell axis was measured to quantify chondrocyte stacking. P, The number of chondrocytes in half of the ethmoid plate was counted (n = 5). Q, The length/width ratio of the chondrocytes in Figure 5A′–N′ was measured (at least 60 cells were measured per group, n = 3). R, The chondrocyte orientation was indicated by rose plot (at least 60 cells were measured per group, n = 3). Orientation was significantly differed from that of control and vehicle control (Watson's U^2^ test; *P < .05, **P < .01). One‐way ANOVA followed by Dunnett's multiple comparison tests were performed for statistical analysis of chondrocyte number and shape (*P < .05, **P < .01). Scale bars: 50 μm in A–N, 5 μm in A′–N′

**FIGURE 5:**
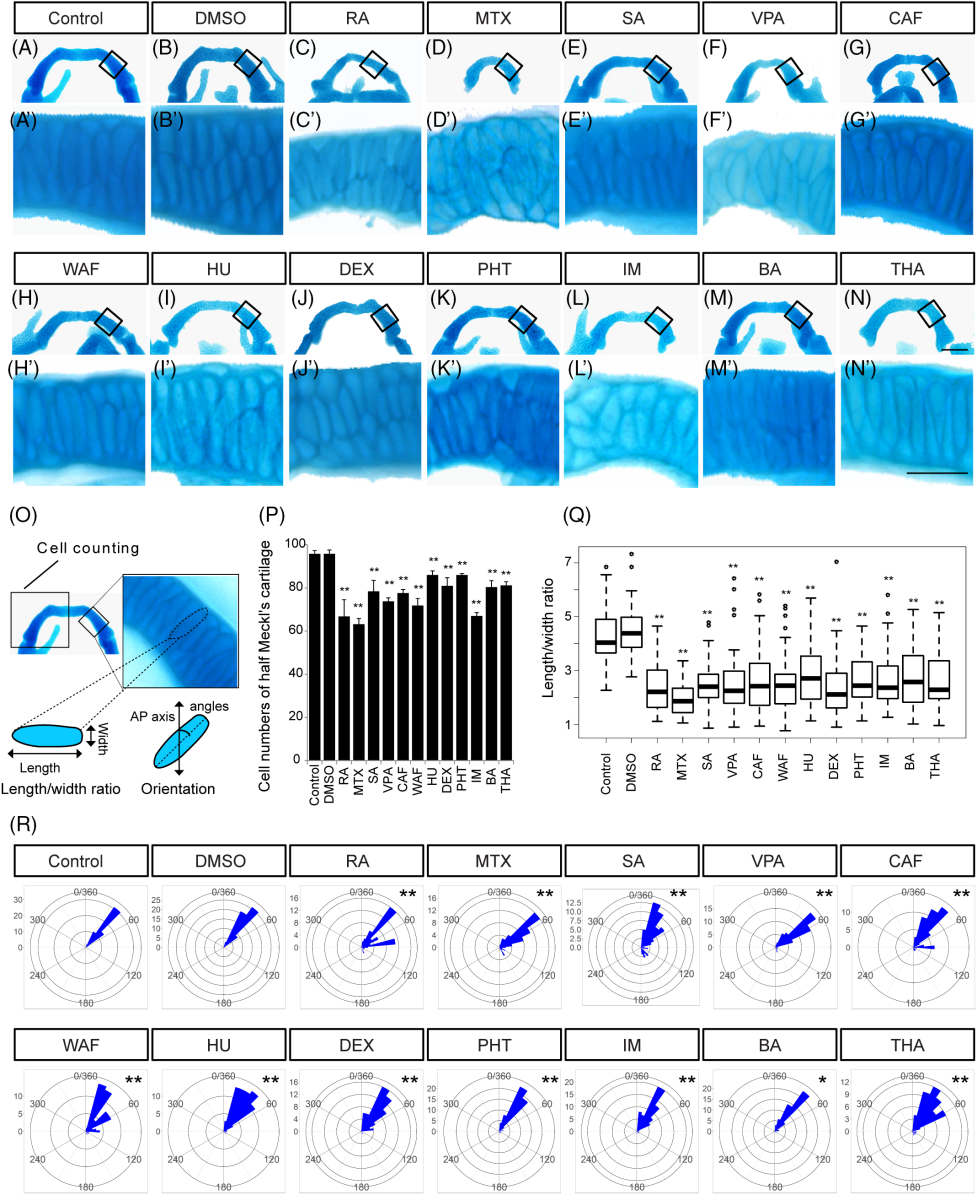
The number of chondrocytes and their shape in the Meckel's cartilage were affected by teratogen treatment. A–N, The Meckel's cartilage was dissected from Alcian blue‐stained samples and was flat‐mounted. Anterior is to the top. A′–N′, Magnified view of chondrocytes in the Meckel's cartilage indicated by the boxed area in A–N. Anterior is to the top. O, The area used for cell counting in the Meckel's cartilage and the definition of the length and width of the chondrocytes used for the cell shape analysis. The orientation of the longest cell axis was measured to quantify chondrocyte stacking. P, The number of chondrocytes in half of the Meckel's cartilage was measured (n = 5). Q, The length/width ratio of the chondrocytes in A′–N′, was measured (at least 60 cells were measured per group, n = 3). R, The chondrocyte orientation was indicated by rose plot (at least 60 cells were measured per group, n = 3). Orientation was significantly different from those of control and vehicle control (Watson's U^2^ test; *P < .05, **P < .01). One‐way ANOVA followed by Dunnett's multiple comparison tests were performed for statistical analysis of chondrocyte number and shape (*P < .05, **P < .01). Scale bars: 50 μm in A–N, 20 μm in A′–N′

We next examined at the cellular level how the malformation of the ethmoid plate and Meckel's cartilage was induced by teratogen treatment. Morphogenesis of the ethmoid plate and Meckel's cartilage is mediated by the convergence and integration of facial prominences, followed by elongation with cell proliferation.^72^, ^73^ In addition to cell proliferation, chondrocyte shaping and stacking are required for proper formation and function of the craniofacial cartilage. We analyzed which of these steps were influenced by teratogen treatment, focusing on the morphology of the ethmoid plate and Meckel's cartilage, and the number of chondrocytes and their morphology (Figures 4 and 5).

The morphology of the ethmoid plate was disturbed by teratogen treatment (Figures 3B,C and 4C–N). RA‐treated embryos displayed an ethmoid plate with a rod‐like shape (Figure 4C). MTX‐, SA‐, VPA‐, CAF‐, WAF‐, HU‐, DEX‐, PHT‐, IM‐, and THA‐treated embryos showed a clefting or rough edge of the anterior ethmoid plate (Figure 4D–L,N). A prominent morphological defect in the ethmoid plate was not observed in BA‐treated embryos (Figure 4M). Mature chondrocytes showed an elongated shape and stacking on each other to form linear columns at 96 hpf in control embryos (Figure 4A′,B′). In teratogen‐treated embryos, chondrocytes showed atypical morphology and lost the stacked structure (Figure 4C′–N′). Rounded chondrocytes were observed in RA‐, MTX‐, SA‐, DEX‐, and THA‐treated embryos (Figure 4C′–E′,J′,N′). In VPA‐, CAF‐, and IM‐treated embryos, chondrocytes showed decreased size (Figure 4F,F′,G,G′,L,L′). In WAF‐treated embryos, chondrocytes showed increased size compared to the control group (Figure 4H′). HU‐, PHT‐, and BA‐treated embryos exhibited smaller chondrocytes with rounded morphology (Figure 4I,I′,K,K′,M,M′).

To obtain quantitative results, the number of chondrocytes in the anterior half of the neurocranium (the ethmoid plate and the trabeculae) was counted and the length/width ratio and the orientation of each chondrocyte in the ethmoid plate were measured (Figure 4O–R). The number of chondrocytes was significantly decreased by all of the teratogen treatments except HU, PHT, and THA treatment (Figure 4P). In order to quantify the disturbance of chondrocyte morphology, the length/width ratio and the orientation of the longest cell axis were measured (Figure 4Q,R). The average ratio was greater than 2 in the control and vehicle control, while the average ratio in all teratogen‐treated groups was significantly decreased (Figure 4Q). The chondrocyte stacking was also significantly disturbed by all of the teratogen treatments (Figure 4R). Thus, the morphological anomalies of the neurocranium induced by teratogen treatment were caused by both a decreased number of chondrocytes and a morphological defect of the chondrocytes.

The same analyses were performed for the Meckel's cartilage in the viscerocranium (Figure 5). The Meckel's cartilage had an inverse U shape in control and vehicle control (Figure 5A,B). After teratogen treatment, the morphology of the Meckel's cartilage was changed and its size was decreased (Figures 3E,F and 5C–N). Mature chondrocytes were elongated and stacked on each other to form a columnar structure at 96 hpf in control and vehicle control (Figure 5A′,B′). Rounded chondrocytes and a lack of stacked structure were observed in the teratogen‐treated embryos (Figure 5C′–N′). In order to obtain quantitative results, the number of chondrocytes in half of the Meckel's cartilage was counted and the length/width ratio and the orientation of the chondrocytes was measured (Figure 5O–R). The number of chondrocytes in the Meckel's cartilage was significantly decreased in all treatment groups; however, no significant difference was observed between the control and the vehicle control groups (Figure 5P). The average length/width ratio was greater than 4 in the control and vehicle control (Figure 5Q). The average length/width ratio was significantly decreased in all treatment groups (Figure 5Q). The chondrocyte stacking in the Meckel's cartilage was also disturbed significantly by all of the teratogen treatments (Figure 5R). Thus, morphological anomalies of the viscerocranium induced by the teratogens were caused by both a decreased number of chondrocytes and morphological defects of the chondrocytes.

Taken together, these results suggested that craniofacial malformation induced by the teratogens was due to defects of not only chondrocyte number but also chondrocyte shape and stacking. These results also led us to speculate that the development of the CNC cell was directly or indirectly perturbed by teratogen treatment.

### 2.3 The expression of CNC‐related genes was perturbed in chemical‐induced craniofacial malformation

**FIGURE 6:**
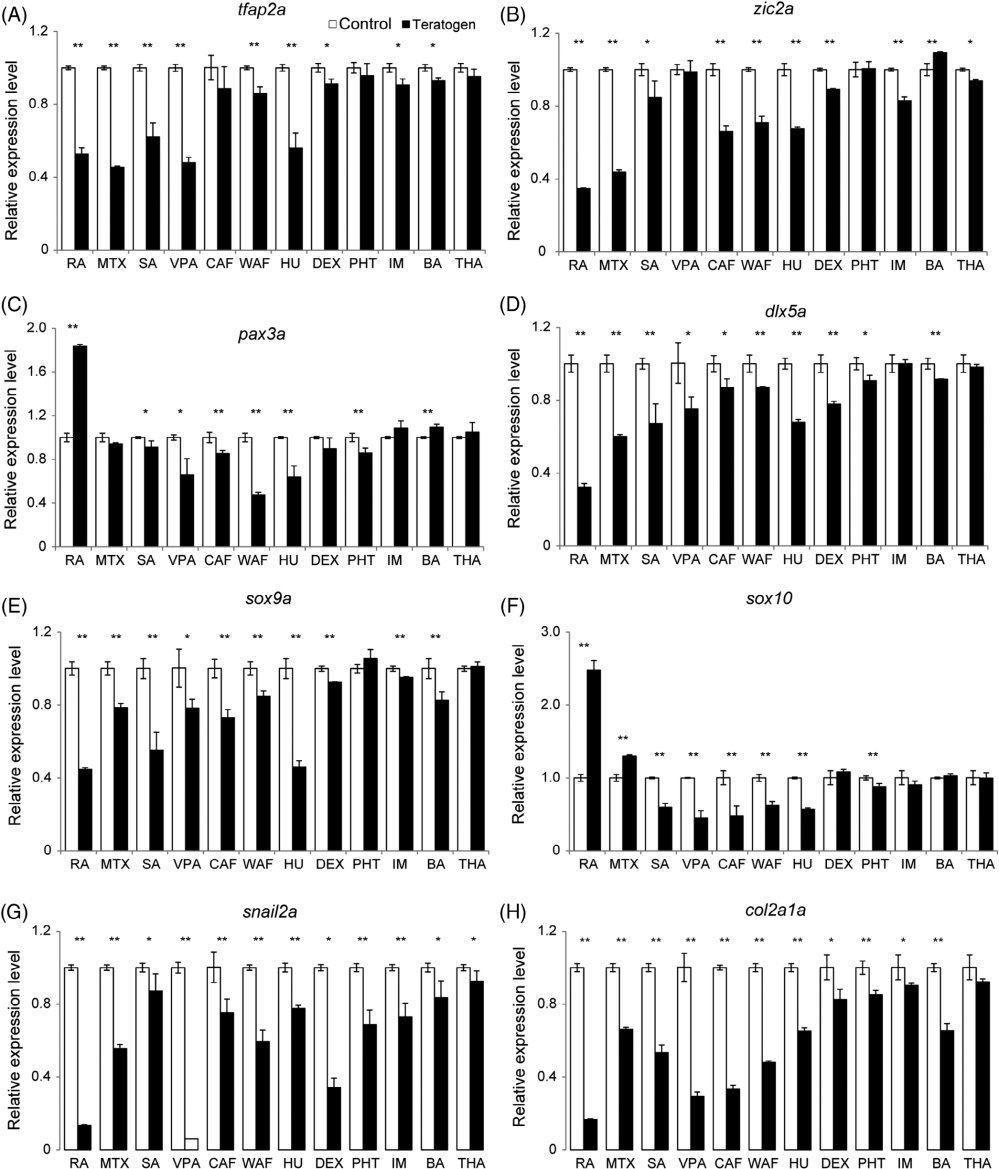
The expression levels of neural crest cell‐related genes were perturbed in teratogen‐treated embryos. Relative expression levels were examined by RT‐qPCR at 48 hpf. The examined genes were as follows: *tfap2a*, A; *zic2a*, B; *pax3a*, C; *dlx5a*, D; *sox9a*, E; *sox10*, F; *snail2a*, G; and *col2a1a*, H. Data are shown as mean ± SD of triplicate samples. Asterisks indicate statistically significant differences between groups (*P < .05, **P < .01, n = 3)

We next investigated whether the observed phenotypes of craniofacial structures and chondrocytes were due to the disruption of CNC development. The expression of CNC‐related genes was analyzed by RT‐qPCR. For this, we selected seven genes that are known to be involved in CNC development and one gene that is a marker of a type of progeny of CNC: *tfap2a*, *zic2a*, *pax3a* and *dlx5a*, *sox9a*, *sox10*, *snail2a* and *col2a1a* (Figure 6). All of these genes are also implicated in human birth defects.^74^, ^75^, ^76^, ^77^, ^78^, ^79^, ^80^, ^81^, ^82^, ^83^ After teratogen treatment, samples were collected at 48 hpf to examine the effect on the CNC development. The expression level of *tfap2a* was significantly decreased in RA‐, MTX‐, SA‐, VPA‐, WAF‐, HU‐, DEX‐, IM‐, and BA‐treated embryos (Figure 6A). Other genes, *pax3a*, *zic2a*, and *dlx5a*, also showed similar effects as *tfap2a*. The expression level of *zic2a* was significantly decreased in RA‐, MTX‐, SA‐, CAF‐, WAF‐, HU‐, DEX‐, IM‐, and THA‐treated embryos (Figure 6B). The expression level of *pax3a* was significantly decreased in SA‐, VPA‐, CAF‐, WAF‐, HU‐, and PHT‐treated embryos, whereas it was increased 1.8‐fold in RA‐treated embryos (Figure 6C). The expression of *dlx5a* was significantly decreased in RA‐, MTX‐, SA‐, VPA‐, CAF‐, WAF‐, HU‐, DEX‐, PHT‐, and BA‐treated embryos (Figure 6D). We also examined the expression levels of *sox9a*, and *sox10* and *snail2a* (Figure 6E–G). The expression level of *sox9a* was decreased significantly by treatment with all teratogens except PHT and THA (Figure 6E). The expression level of *sox10* was also decreased significantly in SA‐, VPA‐, CAF‐, WAF‐, HU‐, and PHT‐treated embryos, whereas it was increased significantly in RA‐ and MTX‐treated embryos (Figure 6F). The expression level of *snail2a* was significantly decreased in all teratogen‐treated embryos (Figure 6G). The expression of *col2a1a* a chondrocyte differentiation marker, was significantly decreased in RA‐, MTX‐, SA‐, VPA‐, CAF‐, WAF‐, HU‐, DEX‐, PHT‐, IM‐, and BA‐treated embryos (Figure 6H). In THA‐treated embryos, the expression level of *col2a1a* was not significantly decreased, although a tendency to decrease was observed. The strength of the effect on the expression levels of the CNC genes observed corresponded to the severity of the phenotypic defect. RA‐, MTX‐, SA‐, and VPA‐treated embryos exhibited absence of cranial structures (Figure 2D–G). In contrast, PHT‐, IM‐, BA‐, and THA‐treated embryos showed minor phenotypes of the ethmoid plate and the Meckel's cartilage, and weaker disturbances of the gene expression levels (Figure 2H–K).

### 2.4 Craniofacial structures such as eyes, otic vesicles, and the heart displayed morphological defects caused by teratogen treatment

The expression levels of CNC‐related genes such as *tfap2a*, *pax3a*, *sox9a*, *sox10*, and *snail2a* were significantly decreased (Figure 6A,C,E–G). These genes have also been reported to be genes responsible for neurocristopathy, a diverse class of disorders such as craniofacial anomalies, ocular defects, hearing disorders and cardiac defects. Our results showed that teratogen‐exposed embryos exhibited malformations in the eyes, otic vesicles, and heart (Figure 1).

**FIGURE 7:**
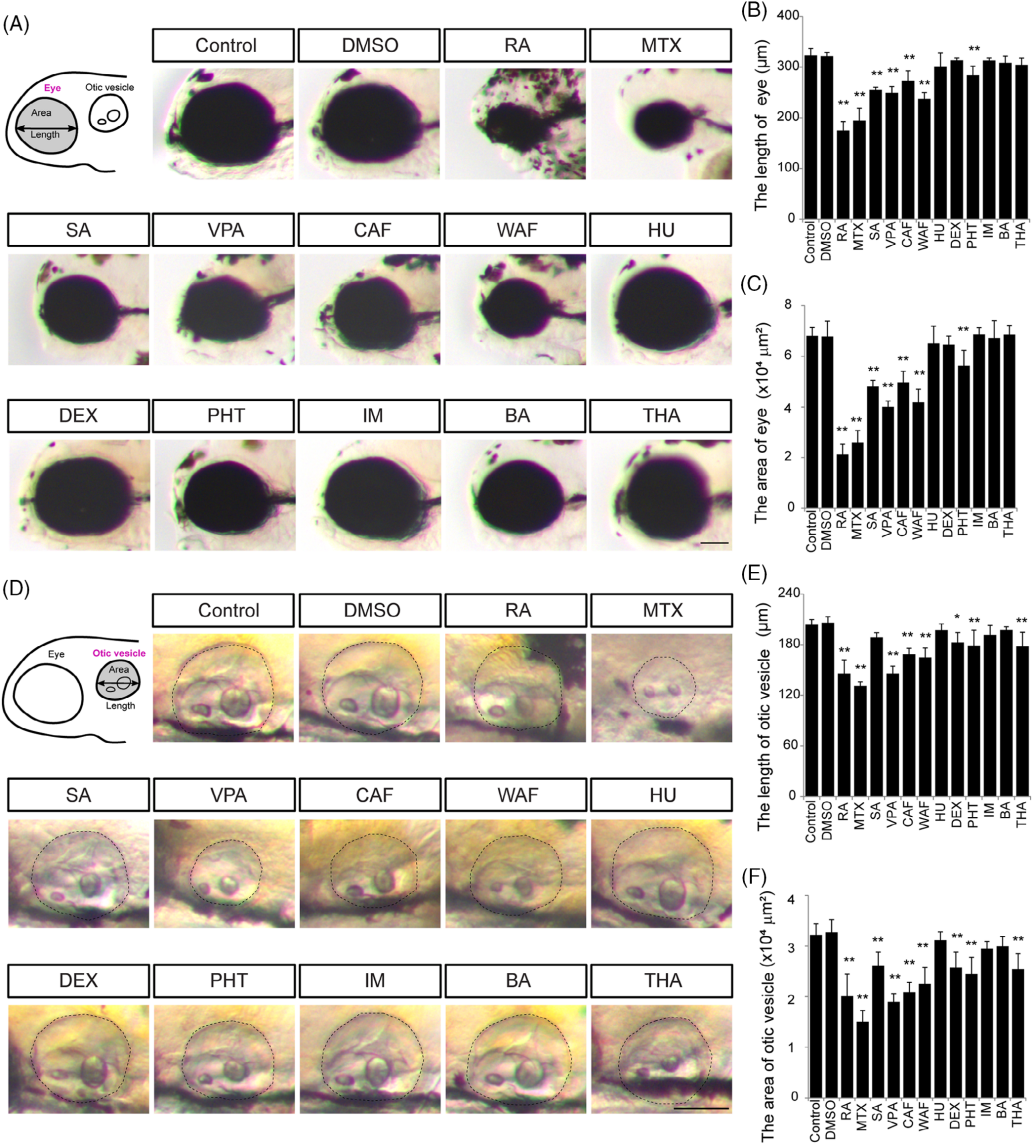
Defects in eye and otic vesicle were observed in teratogen‐treated embryos. A, Schematic image and bright field images of the eye. Anterior is to the left. B and C, Quantification of the length and area of the eye. D, Schematic image and bright field images of the otic vesicle. E and F, Quantification of the length and area of the otic vesicle. Asterisks indicate statistically significant differences between groups (*P < .05, **P < .01, n = 5). Scale bars: 100 μm

**FIGURE 8:**
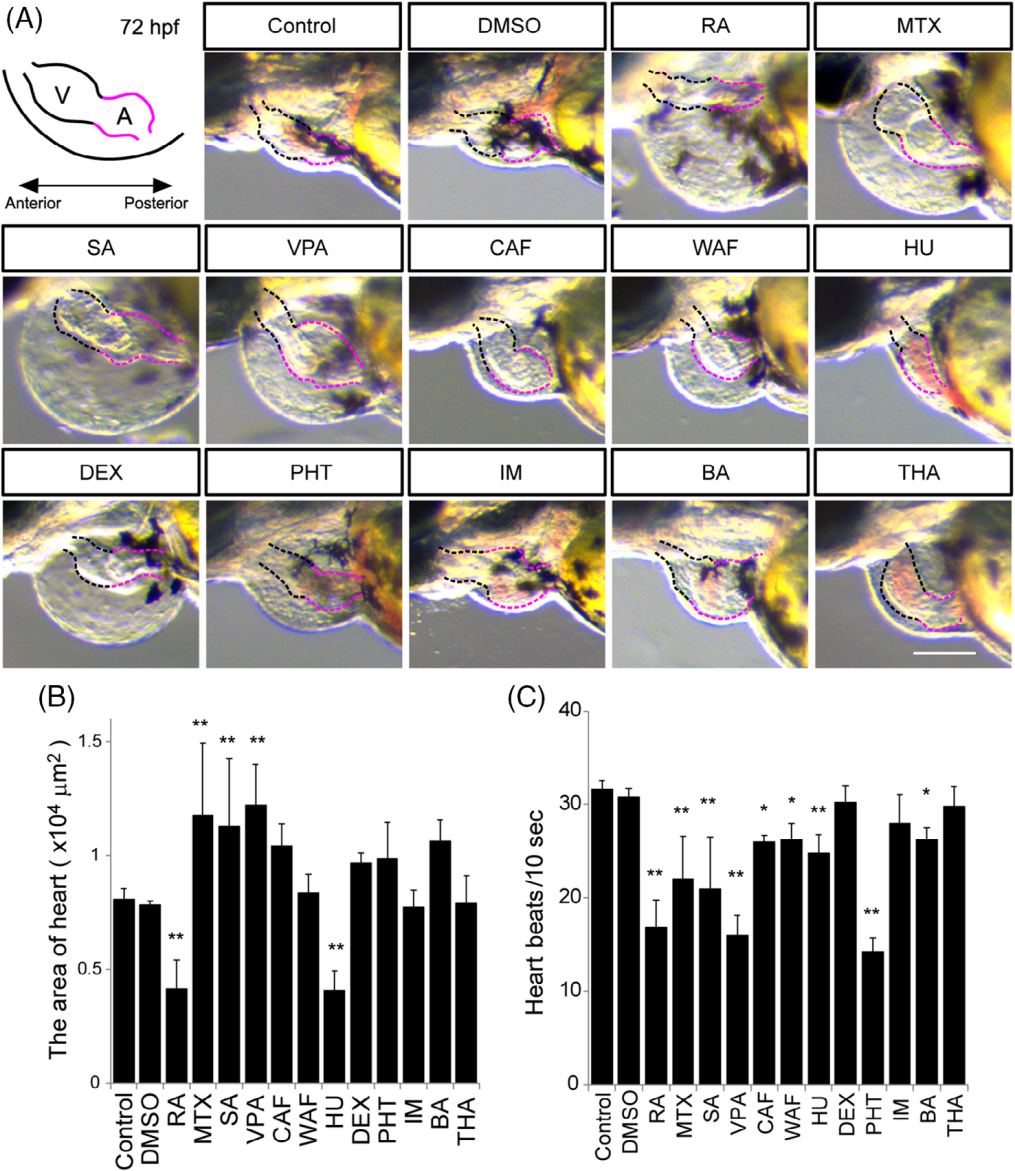
Morphological defects of the heart and measurement of the heart beat rate. A, Illustration of the embryonic heart at 72 hpf. V stands for the ventricle marked by the black‐dotted line. A stands for the atrium marked by the red‐dotted line. B, The maximum ventricle ventricular and maximum atrium diastole were measured. C, Heart beat rate was calculated in a 10‐second window based on supplemental [Link], [Link]. Asterisks indicate statistically significant differences between groups (*P < .05, **P < .01, n = 5). Scale bar: 100 μm

Thus, we next performed quantification of the severity of defects in the eyes, otic vesicles, and heart (Figures 7 and 8). To assess the degree of eye defects, the length and area of the eye were measured at 96 hpf (Figure 7A). In control and vehicle control, the average length of the eye was greater than 300 μm and the average area of the eye was greater than 6.0 × 10^4^ μm; however, the average length and area were significantly decreased in RA‐, MTX‐, SA‐, VPA‐, CAF‐, WAF‐, and PHT‐treated embryos (Figure 7B,C).

The degree of the otic vesicle anomaly was assessed by measuring the length and area of the otic vesicle (Figure 7D). In control and vehicle control, the average length of the otic vesicle was greater than 200 μm, and the average area of the otic vesicle was greater than 3.0 × 10^4^ μm; however, the average length and area were significantly decreased in RA‐, MTX‐, VPA‐, CAF‐, WAF‐, DEX‐, PHT‐, and THA‐treated embryos (Figure 7E,F). Teratogen‐treated embryos also showed morphological defects in otoliths, but the number of otoliths was not affected (data not shown).

**FIGURE 9:**
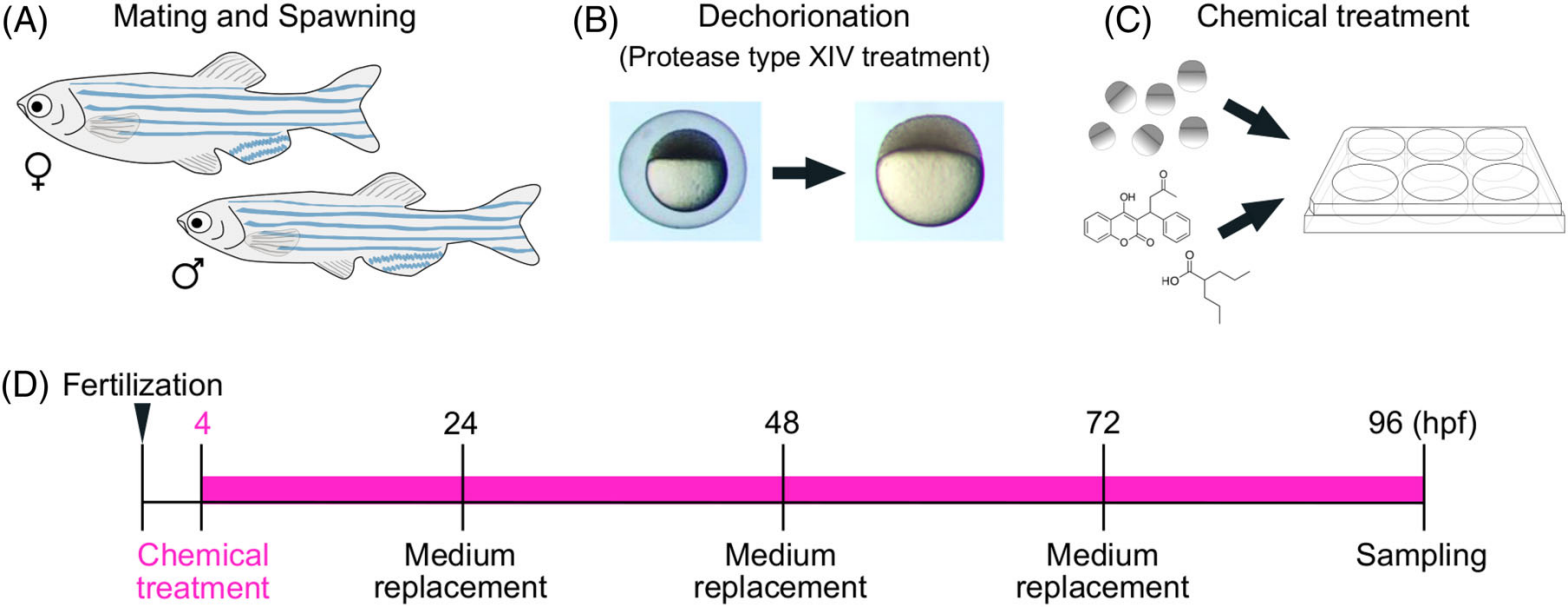
Chemical exposure procedure. A, Adult male and female zebrafish were placed together in a breeding tank equipped with a partition plate and a mesh tray for collecting fertilized eggs the day before spawning. B, Before chemical exposure, fertilized eggs were treated with protease type XIV for dechorionation. C, The dechorionated eggs were placed in a 6‐well plate and treated with a teratogen. D, Time course of chemical treatment. Chemical treatment started at 4 hpf and exposure medium was replaced daily. The samples were collected at 96 hpf and were processed for further analysis

To investigate heart abnormalities, the heart morphology and movement were examined at 72 hpf (Figure 8 and [Link], [Link]). The zebrafish heart consists of an atrium and a ventricle. After teratogen treatment, morphological defects in the atrium and ventricle were observed (Figure 8A). The heart size, which is the size of the atrium plus the ventricle, was measured (Figure 8B). Hyperplasia of the atrium and the ventricle were observed in MTX‐, SA‐, and VPA‐treated embryos (Figure 8A,B and [Link], [Link]). Although a significant difference was not observed in CAF‐, DEX‐, PHT‐, or BA‐treated embryos, a tendency of hyperplasia was observed (Figure 9B and [Link], [Link], [Link], [Link]). Hypoplasia of the atrium and the ventricle were observed in RA‐ and HU‐treated embryos (Figure 8A,B and [Link], [Link]). To examine the relationship between the morphological changes of the heart and its function, the heartbeat rate was measured (Figure 8C and [Link], [Link]). In accord with the heart morphological defects, the heartbeat rate was significantly decreased in RA‐, MTX‐, SA‐, VPA‐, CAF‐, HU‐, PHT‐, and BA‐treated embryos (Figure 8C and [Link], [Link], [Link], [Link]). A significant morphological difference was not observed in CAF‐ or WAF‐treated embryos; however, a functional heart defect was observed in these embryos (Figure 8B,C). The dimensions of the morphological changes found in eye, ear, and heart were also greater than those of the changes in overall size of the embryos (Figure 2Q).

Taken together, our results indicated that there were specific teratogen‐induced craniofacial defects as well as heart defects, suggesting that these individuals were a phenocopy of neurocristopathy.

## 3 DISCUSSION

In this present study, we addressed two questions as follows: (a) Do teratogens which induce craniofacial anomalies in mammals cause the same anomalies in zebrafish? (b) Do teratogens target the development of CNC cells, leading to craniofacial anomalies in zebrafish? Our findings provided three novel messages. (a) Twelve teratogens that cause craniofacial abnormalities in mammals were also found to disturb craniofacial development in zebrafish. (b) The craniofacial anomalies occurred due to a decrease in the number of chondrocytes and to disturbing the chondrocyte size and stacking, as well as affecting CNC numbers. (c) Our results showed an association between craniofacial defects induced by teratogens and neurocristopathy, and indicated that zebrafish is a suitable model animal for studying environmental risk factors for neurocristopathy.

### 3.1 Teratogens act as environmental factors that disrupt the development of NC cells and chondrocyte differentiation and maturation, leading to craniofacial malformation

Craniofacial morphogenesis involves a complex series of developmental events that ultimately create diverse facial morphologies in vertebrates. Despite the differences of vertebrates' craniofacial structures, the NC cell and its roles in craniofacial development are conserved across vertebrates.^3^, ^84^, ^85^ The etiology of craniofacial anomalies involves the interplay between genetic factors and environmental factors.^2^ Extensive knowledge about various teratogens that induce craniofacial defects in mammals, including humans, has been reported (Table 1). The teratogens used in our experiments are well known to induce multiple craniofacial disorders, such as micrognathia, cleft palate, microcephaly, eye defects, and ear abnormalities based on epidemiological data and experiments in mammals.^39^, ^41^, ^43^, ^45^, ^47^, ^61^, ^63^, ^64^, ^86^, ^87^ However, there is limited information about the cellular and molecular mechanisms of craniofacial malformations caused by teratogens.

Here, we exposed zebrafish embryos to specific teratogens. In accord with reports of studies in mammals, our results showed that the teratogens tested here clearly induced malformation of the neurocranium and viscerocranium (Figures 2 and 3). These results led us to speculate that CNC development in zebrafish is severely impaired by these chemicals. In accord with this possibility, RA, MTX, VPA, CAF, and DEX have been reported to affect CNC development in mammals and other vertebrates.^88^, ^89^, ^90^, ^91^, ^92^, ^93^ The other chemicals that we tested, namely SA, WAF, HU, PHT, IM, BA, and THA, have been reported to induce craniofacial malformations in mammals, but there have been no reports about the cellular and molecular mechanisms of these malformations.^39^, ^41^, ^45^, ^47^, ^59^, ^63^, ^94^

In the present study, we showed that all of these teratogens affected craniofacial morphogenesis as well as CNC development at the cellular level. Our large‐scale analysis of numerous teratogens revealed that they caused aberrant CNC development and led to craniofacial malformation. Thus, zebrafish and mammals share similar responses to various chemicals regarding the chemically induced craniofacial anomalies. We showed that 12 chemicals impaired CNC development in zebrafish; however, whether these chemicals affect CNC development directly or indirectly needs to be examined in future studies.

Furthermore, we found that the teratogens also targeted chondrocyte number, shape and stacking. During the process of chondrocyte differentiation and maturation, the cellular features of chondrocytes progressively change within the growth plate in craniofacial cartilage. Small, round chondrocytes need to undergo proliferation and elongation to give rise to long, flattened chondrocytes, and finally become stacked in longitudinal columns in mammals.^95^ Zebrafish cartilage formation occurs through the same process, which is thought to be evolutionally conserved among vertebrates.^96^ Most of the craniofacial cartilage elements are formed by 96 hpf in zebrafish, and the chondrocytes of the neurocranium and viscerocranium, such as those in the ethmoid plate and Meckel's cartilage, show characteristic stacking and organization of thin, elongated chondrocytes that are assembled on their respective cartilage element.^97^, ^98^ Craniofacial malformation can also be caused by genetic mutations in NC‐derived chondrocytes in mammals and zebrafish.^99^, ^100^ Round‐shaped chondrocytes and loss of columnar structure are typical phenotypes caused by such mutations.^98^, ^101^, ^102^

Intriguingly, the defects caused by genetic mutations were phenocopied by teratogen treatments in our experiments (Figures 4 and 5). We focused on the ethmoid plate and Meckel's cartilage because these two structures are derived from the CNC population, as proven by lineage tracing experiments.^25^, ^71^, ^103^, ^104^ As we expected, the number of cartilage cells in the ethmoid plate and Meckel's cartilage was decreased by the teratogen treatment. This suggests that chondrocytes in both of these cranial structures probably cease proliferation or undergo increased apoptosis. Such a defect was confirmed by the decreased expression level of *col2a1a*, a differentiation marker of chondrocytes (Figure 6H). Therefore, our results indicate that the teratogens tested here directly or indirectly block the development of CNC cells and chondrocytes, leading to craniofacial malformation.

Our results support the possibility that various chemical‐induced craniofacial abnormalities share similar cellular mechanisms via aberrant development of CNC in zebrafish and mammals. Our results demonstrate a similar response to the teratogens at the cellular levels; however, the key signaling pathways and detailed mechanism disrupted by these teratogens will need to be elucidated to clarify the basis of our findings.

### 3.2 Zebrafish is a powerful model for investigating gene‐environment interactions causing craniofacial malformation

It has been thought that gene‐environment interactions increase the risk of craniofacial anomalies.^2^ However, there are relatively few reports about the detailed mechanisms of interaction between genetic variations and environmental factors due to limited animal models. Here, we showed that zebrafish share conserved responses to teratogens with mammals, suggesting that zebrafish is an appropriate model for investigating the contribution of an environmental factor to craniofacial disorders. Neurocristopathies are human diseases associated with abnormal development of NC cells. These diseases are thought to be consequences of defects in NC cell specification, migration, proliferation and/or differentiation. The genes responsible for neurocristopathies have been identified in the cases of several clinical abnormalities, as follows: *tfap2a* (Branchio‐oculo‐facial syndrome), *zic2a1* (holoprosencephaly), *pax3*, *sox10*, and *snail2* (Waardenburg syndrome and Hirschsprung's disease), and *sox9* (Pierre Robin sequence).^74^, ^75^, ^80^, ^81^, ^83^, ^105^ Mutations in *tfap2a* result in Branchio‐oculo‐facial syndrome, characterized by cleft palate‐craniofacial disorder.^80^ In zebrafish, the *tfap2a* mutant *lockjaw* shows severe defects in the cartilage of both the neurocranium and viscerocranium.^106^ *Pax3* is required for the development of multiple NC lineages and is implicated in NC disorders observed in humans, including Waardenburg syndrome, which results in hearing defects and craniofacial anomalies.^107^, ^108^ Mutations in *sox9* are reported in Pierre Robin sequence, which is characterized by micrognathia, glossoptosis, and cleft palate.^74^, ^79^ Similar symptoms are also observed in zebrafish *sox9* mutants.^109^ These reports indicate that mutations of the genes responsible for neurocristopathy cause similar phenotypes, such as micrognathia, microcephaly, eye defects, abnormal ears, and heart defects, in fish and humans.

The NC development is also disrupted by environmental factors such as alcohol and cigarette smoking, which are thought to lead to neurocristopathies.^10^ However, few environmental factors have been proven to disrupt NC development. The mechanism of such disruption by various chemicals needs to be examined.^10^ Interestingly, zebrafish with mutations of genes responsible for human neurocristopathy mimic the phenotypes in humans, such as micrognathia, microcephaly, eye defects, abnormal ears, and heart defects. Here, our results showed disturbed expression levels of the responsible genes, *tfap2a*, *pax3*, *sox9*, *sox10*, and *snail2* after specific teratogen treatments (Figure 6A,C,E–G). Furthermore, besides craniofacial malformation, these teratogen‐treated animals displayed eye and ear defects and heart malformations (Figures 7 and 8). These results demonstrate that chemical‐induced craniofacial malformation resembles neurocristopathy in zebrafish even though the teratogens do not alter the genomic sequence. Besides the responsible genes for neurocristopathy, it is known that mutations in *dlx5a* cause split‐hand/ft malformation 1 with sensorineural hearing loss (SHFM1D) and mutations in *col2a* cause Kniest dysplasia and Stickler syndrome.^78^, ^82^ Teratogen‐treated zebrafish also partly displayed the similar phenotypes to those found in patients with SHFM1D, Kniest dysplasia and Stickler syndrome. The expression level of the responsible genes was decreased (Figure 6D,H). This evidence leads us to speculate that teratogen‐induced alteration of the transcription of responsible genes also leads to craniofacial anomalies. Thus, zebrafish provide a new platform for investigating gene‐environment interactions. We can create in zebrafish heterozygous mutations of disease‐related genes or genes identified from patients and expose the mutant zebrafish to chemicals to analyze the mechanisms of gene‐environment interactions. Furthermore, our results indicate that certain teratogens that are prescription medicines have the potential to increase neurocristopathies, in addition to demonstrating the usefulness of zebrafish as a craniofacial disease model.

Our results also suggest the vulnerability or sensitivity of CNCs to teratogens. Ribosome biogenesis is thought to have an essential role in skeletal development and the pathogenesis of congenital skeletal anomalies.^110^ Dixon et al showed that ribosome biogenesis has a pivotal role in CNC formation and craniofacial abnormalities.^28^ Also, alcohol was reported to target ribosome biogenesis and lead to craniofacial defects.^111^ Although the molecular nature of CNC vulnerability could conceivably be ribosome biogenesis in CNCs, further research will be needed to examine the molecular target(s) of each teratogen to clarify the basis of the vulnerability of CNCs to teratogens.

## 4 EXPERIMENTAL PROCEDURES

### 4.1 Zebrafish strain and maintenance

Wild‐type zebrafish, *D. rerio*, with the RIKEN WT genetic background were maintained with a 14‐hours light/10‐hours dark cycle. Water temperature was controlled at 28 (±1)°C, and water quality conditions were maintained according to The Zebrafish Book^112^ and the Guide for the Care and Use of Laboratory Animals, eighth edition.^113^

### 4.2 Test chemicals

The test chemicals used in this study are listed in Table 1. Before embryo exposure experiments, a dose range‐finding study was performed according to Figure 9. Four different concentrations, 1, 10, 100, and 1 mM, of each chemical were used in the dose range‐finding study. The highest soluble concentration was determined as the highest concentration at which precipitation did not occur. After the dose range‐finding study, the exposure concentration was determined with a common dilution ratio of the highest concentration into E3 medium of 2 or 3. The exposure concentration was determined as the concentration at which embryos were 100% viable and displayed the most severe and frequent phenotype. The exposure concentrations used in the exposure experiments were as follows: retinoic acid (5 nM), methotrexate (200 μM), salicylic acid (400 μM), valproic acid (30 μM), caffeine (500 μM), warfarin (60 μM), hydroxyurea (1 mM), dexamethasone (200 μM), phenytoin (200 μM), imatinib (250 μM), boric acid (1 mM), and thalidomide (400 μM). Other publications regarding zebrafish toxicity tests were also referred to in order to determine the appropriate exposure concentrations.^114^, ^115^, ^116^, ^117^, ^118^, ^119^, ^120^, ^121^, ^122^

### 4.3 Egg production and embryo exposure

Adult male and female zebrafish were housed separately prior to spawning and placed together in a breeding tank equipped with a mesh tray the day before spawning. Spawning was stimulated in the morning when the light was turned on, and fertilized eggs were collected within 1 hour after the light stimulation (Figure 9A). Before the exposure experiments, the fertilized eggs were rinsed with E3 medium (5 mM NaCl, 0.17 mM KCl, 0.33 mM CaCl_2_, 0.33 mM MgSO_4_) and incubated with E3 medium containing 1 mg/mL Protease type XIV (Sigma‐Aldrich, MO, USA) for 10 minutes at room temperature for dechorionation (Figure 9B). Embryos were washed several times with E3 medium to remove the chorions; then, the dechorinated embryos were incubated in E3 medium for 1 to 2 hours until exposure experiments. Embryos were exposed to a test chemical solution at the concentrations listed in Table 1 in E3 medium at 4 hpf according to the conditions reported by Kimmel et al and incubated until 96 hpf^123^ (Figure 9C). The test chemical solution was replaced daily and samples were collected at 96 hpf, when basic craniofacial morphology is formed (Figure 9D). All experiments were conducted in triplicate, and at least 30 embryos were investigated per treatment.

### 4.4 Morphological observation and quantification

Morphological changes in the eyes, otic vesicle, and heart were observed under a stereomicroscope at 96 hpf. To quantify the size of the eyes and otic vesicle, images were captured with a Leica M80 equipped with a DFC450C (Leica, Wetzlar, Germany). Then, images were analyzed using ImageJ (National Institutes of Health, MD, USA). For heart size measurement, the maximum ventricular diastole and the atrium diastole were captured using movies and analyzed. Heart rate was analyzed using movies made with bright‐field microscopy, and the number of heart beats in a 10‐seconds window were recorded.

### 4.5 Cartilage staining

Zebrafish embryos were fixed at 96 hpf with 4% paraformaldehyde in phosphate‐buffered saline for 2 hours. After the samples were washed with 100 mM Tris (pH 7.5), they were treated with 100 mM Tris (pH 7.5) containing 25 mM MgCl_2_ for 30 minutes. Then, samples were transferred into a 0.05% Alcian blue (MP Biomedicals, CA, USA) dissolved in 80% ethanol (EtOH), 100 mM Tris (pH 7.5), and 25 mM MgCl_2_ overnight. Next, the stained samples were placed in 80% EtOH containing 100 mM Tris (pH 7.5) and 25 mM MgCl_2_ and then gradually rehydrated by incubating with 50% and 25% EtOH in 100 mM Tris (pH 7.5) for 30 minutes per step. Samples were washed with potassium hydroxide (KOH) for 30 minutes and processed to remove pigmentation by bleaching in 3% hydrogen peroxide and 0.1% KOH for 30 minutes. Then, samples were rinsed with 35% saturated sodium borate decahydrate (NaBO_4_) for 30 minutes. For clearing, the samples were treated with 1% trypsin (Wako, Osaka, Japan) dissolved in 35% saturated NaBO_4_ for 30 minutes and then washed with 0.5% KOH overnight. All procedures were performed at room temperature.

### 4.6 Chondrocyte measurement

After cartilage staining, samples were transferred into 100% glycerol. The craniofacial structure was disassembled into the neurocranium and viscerocranium using fine forceps. Each structure was mounted on a glass slide, and the mounted samples were covered with glass coverslips and pressed to prepare flat‐mount samples. For preparation of flat‐mounted samples, the stained samples were placed on a glass slide and were slowly covered with a cover slip placed by using forceps to minimize the generation of air bubbles. Glycerol was added to fill the space between the coverslip and the glass slide. Photographs were then taken using a BZ‐710 microscope (Keyence, Osaka, Japan). The number of chondrocytes in the ethmoid plate of the neurocranium and Meckel's cartilage of the viscerocranium was counted. Chondrocyte stacking analysis was performed as previously described.^102^, ^124^ The angle between the anteroposterior axis and the longest cell axis was measured and plotted as a rose plot. For statistical analysis, Watson's U^2^ tests were performed using RStudio (RStudio Inc., MA, USA). For length and width measurement and stacking measurement, more than 60 chondrocytes were measured using ImageJ. All measurements were performed in triplicate.

### 4.7 Real‐time quantitative RT‐qPCR analysis

Thirty zebrafish embryos at 48 hpf were homogenized in TRIzol reagent (Invitrogen, CA, USA). Total RNA was purified with an RNeasy Mini Kit (Qiagen, Hilden, Germany) according to the manufacturer's protocol. The purified RNA with an absorbance ratio of 1.8 to 2.0 at A260/280 was used for further analysis. Total RNA (1.5 μg) was used for reverse transcription using the SuperScript III first‐strand synthesis system (Thermo Fisher Scientific, MA, USA). RT‐qPCR was performed with five‐fold diluted complementary DNA, Taqman Master Mix (Thermo Fisher Scientific, MA, USA), TaqMan probes, and gene‐specific primers (Bio‐Rad, CA, USA) using the 7500 Fast Real‐Time PCR System. The housekeeping gene *glyceraldehyde‐3‐phosphate dehydrogenase* (*GAPDH*) was used as an internal control. The gene‐specific primers were as follows: *tfap2a*, *zic2a*, *pax3a*, *dlx5a*, *sox9a*, *sox10*, *snail2a*, and *col2a1a* primers. The expression levels of target genes were normalized by the *GAPDH* expression level, and the normalized relative gene expression was calculated using the comparative Ct (2^−ΔΔCt^) method. All reactions were performed in triplicate.

### 4.8 Statistical analysis

Statistical analyses were performed using GraphPad prism version 8 for Windows (La Jolla, CA, USA), and boxplot and rose plot graphs were made using RStudio (RStudio Inc., MA, USA). *P*‐values were calculated with one‐way ANOVA followed by Dunnett's multiple comparison tests. *P*‐values <.05 were considered statistically significant. All data are presented as the mean ± SD unless otherwise specified.

## AUTHOR CONTRIBUTIONS

**Shujie Liu:** Conceptualization; data curation; formal analysis; investigation; methodology; validation; visualization; writing‐original draft; writing‐review and editing. **Rika Narumi:** Data curation; methodology; validation; visualization; writing‐original draft; writing‐review and editing. **Naohiro Ikeda:** Writing‐original draft. **Osamu Morita:** Writing‐original draft. **Junichi Tasaki:** Conceptualization; data curation; methodology; project administration; supervision; validation; writing‐original draft; writing‐review and editing.

## Supporting information


**Movie S1** Heat beats of control embryo at 72 hpf. Samples were embedded in 3% methylcellulose and were recorded under a stereomicroscope.

**Movie S2** Heart beats of DMSO (vehicle control).

**Movie S3** Heart beats of RA‐treated embryo.

**Movie S4** Heart beats of MTX‐treated embryo.

**Movie S5** Heart beats of SA‐treated embryo.

**Movie S6** Heart beats of VPA‐treated embryo.

**Movie S7** Heart beats of CAF‐treated embryo.

**Movie S8** Heart beats of WAF‐treated embryo.

**Movie S9** Heart beats of HU‐treated embryo.

**Movie S10** Heart beats of DEX‐treated embryo.

**Movie S11** Heart beats of PHT‐treated embryo.

**Movie S12** Heart beats of IM‐treated embryo.

**Movie S13** Heart beats of BA‐treated embryo.

**Movie S14** Heart beats of THA‐treated embryo.
